# Effects of Workplace Gossip on Employee Mental Health: A Moderated Mediation Model of Psychological Capital and Developmental Job Experience

**DOI:** 10.3389/fpubh.2022.791902

**Published:** 2022-04-12

**Authors:** Sheng Cheng, Chien-Chih Kuo, Huai-Chieh Chen, Mei-Chi Lin, Vincent Kuo

**Affiliations:** ^1^Department of Psychology, National Chengchi University, Taipei, Taiwan; ^2^School of Business, Huaiyin Institute of Technology, Huaiyin, China; ^3^Department of Entrepreneurship, Marketing and Management Systems, Nottingham University Business School China, Ningbo, China

**Keywords:** workplace gossip, psychological capital (PsyCap), developmental job experience, mental health, mediated moderation model

## Abstract

Research has demonstrated the effects of workplace gossip on employees' work attitudes and behaviors. However, little emphasis has been placed on the psychological influence of workplace gossip on employees. The present study investigated the relationships among workplace gossip, psychological capital, and individual mental health. Data were collected in three waves from 222 full-time employees of a Taiwanese tourism company to explore the effect of workplace gossip on employees' mental health. The results suggested that workplace gossip was associated with employees' mental health through psychological capital. Moreover, developmental job experience plays a moderator role in the relationships among workplace gossip, psychological capital, and mental health. A moderated mediation model was also proposed in this study.

## Introduction

Workplace gossip (WG) is a frequent occurrence in organizations (1). An employee “producing, listening to, or otherwise participating in evaluative comments” of work-related issue about an absent person would be classified as a WG participant (2). Specifically, researchers have classified WG into two types (1, 3): workplace positive gossip (WPG) and workplace negative gossip (WNG). Participating in these two types of WG can have opposite effects on employee work attitudes, work behaviors, and work outcomes (4). For example, engaging in WPG is positively related to organizational citizenship behaviors (3) and negatively associated with gossiper's employee cynicism (2), whereas participating in WNG may decrease gossipers' work-related in-role performance and job-related well-being (3) or increase employee deviance workplace behaviors (3) and cynicism toward organization (2). Although prior studies have identified WG as a crucial factor that could significantly influence employee work-related attitudes and outcomes in the workplace, little is known about the psychological influences and processes of WG on employees. The present study was designed to uncover the effects and psychological mechanisms of WG on employee mental health (MH).

We sought to explain the relationship between WG and individual MH by employing psychological capital theory, in contrast to previous studies that have shown scant interest in WG as a psychological resource. The psychological capital theory argues that psychological capital (PsyCap) is a type of psychological resource that determines individuals' psychological well-being and behaviors (5). Previous work suggested that WG could be a social cue for an individual to shape the meaning of social information concerning their experiences in specific environments (1, 6), which implies WG could be regarded as a personal resource that individuals use to understand the work environment. Furthermore, cognitive appraisal theory (7) suggests that these personal resources may trigger psychological states of emotion. Thus, the present study tested the hypothesis that WG is a type of social cue implying personal psychological resource consumption when interpreting social information (6), and resource obtainment when exchanging social information (8). When an employee participates in WG, PsyCap dynamics may be influenced, which then affects individuals' MH.

Few studies have investigated the moderating effect of different variables on the relationship between WG and outcomes. Here we probed the boundary effect of developmental job experience (DJE), which refers to self-development opportunities at work (9). Prior studies have demonstrated that individuals with DJE are more sensitive to social information and interpret social cues from a more systematic perspective (10), which may interact with WG interpretation. Specifically, employees with high DJE would be better able to comprehend the social cues of WG than those employee with low DJE. Thus, we hypothesized that DJE could be a key moderating factor, which suggests there is a practical implication for organizations to strengthen the positive effects of WG on employee PsyCap and MH (through PsyCap) or counteract negative effects by helping employees to cope with WG.

## Theory and Hypotheses

### Relationship Between Workplace Gossip and Mental Health

WG is defined as idle talk that involves the exchange of personal information and judgments about colleagues who are not present (11). It is considered a type of social interaction and a source of social information (2). Previous research categorized WG into two types (1, 3). WPG refers to positive statements about an absent colleague, such as their achievements, promotions, or receipt of praise from supervisors. Conversely, WNG is negative statements about the gossip target, such as their deviant behaviors, demotions, or inadequate work abilities. Engaging in WPG and WNG leads to different employee reactions and outcomes in organizations. For instance, Kuo et al. (1) suggested that employees engaging in WPG are exposed to more positive social cues, thereby facilitating their psychological attachment and considerably reducing workplace cynicism. In contrast, employees engaging in WNG experience an unpleasant atmosphere and decreased psychological attachment, which in turn, increases the frequency and tendency of employee cynicism. Therefore, the two types of WG can shape different employee attitudes toward the organization.

Studies have identified WG as channels of informal communication (2) for casual or unconstrained social information (12), even if the information is inaccurate or incomplete. Thus, gossip fulfills a social function by creating close bonds and enforcing workplace norms (3). Moreover, Kuo et al. (1) identified WG as a type of social information that conveys social cues to individuals. WG participants receive specific social cues about their discussion targets when they gossip. These social cues influence an individual's attitude, behavior, and needs through construction of meaning with socially acceptable reasoning, which can provide information on salient expectations and logic (13). Cognitive appraisal theory suggested that the personal mental state of emotion is affected by appraising or evaluating received information (7, 14, 15). Following this reasoning, gossipers engaging in WPG are exposed to positive social cues; gossipers who are interpreting, appraising, or evaluating these social cues may positively enhance their mental states of emotion, feelings, attitudes, and behaviors at work. For example, when employees receive positive social cues, they exhibit higher psychological affect and more positive emotions (16), which may increase their positive MH (17). Conversely, WNG participants receive negative social cues of deleterious information that may have adverse psychological effects. That is, gossipers may negatively interpret, appraise, or evaluate these social cues when participating in WNG, which may exacerbate their mental states. Researchers demonstrated that individuals exposed to negative social cues experience higher work strain, stress, and depression (18) and have lower psychological well-being (19) in the workplace, and this could negatively affect their MH.

When employees engage in WG, specific social cues affect their MH. However, positive gossip implies more affirming social cues, so employees may receive more positive information that improves their MH. Conversely, WNG delivers deleterious information, resulting in more negative social cues and information that adversely affect MH. Accordingly, the following are proposed in this paper:

*Hypothesis 1a*: Participating in WPG is positively correlated with gossiper's MH.
*Hypothesis 1b*: Participating in WNG is negatively correlated with gossiper's MH.

### Relationship Between Workplace Gossip and Psychological Capital

PsyCap is defined as a positive psychological state that helps achieve positive organizational behaviors with four dimensions: self-efficacy, hope, optimism, and resilience (5). In the context of cognitive appraisal theory, the social cues of WG may affect the personal positive psychological state of PsyCap by interpreting, appraising, or evaluating the relevant information (15). In addition, PsyCap can also be regarded as a psychological resource (20) that provides a competitive advantage and a subjective sense of well-being (5). Following this logic, WG serves as a kind of social cue that requires psychological resources to interpret, but it also shapes an individual's sense of reality and their perception of the meaning of individuals or organizations (1). Thus, psychological resources are spent to interpret, attribute, and cope with social cues from WG (21). WG is also a way to obtain social information and exchange social resources (22) that individuals use to facilitate personal capabilities in social systems such as workplaces (8). In other words, WG both provides social cues that individuals can leverage to gain social resources from information exchange and simultaneously depletes psychological resources to interpret them. Accordingly, this article proposes that there is a dynamic relationship between WG and PsyCap from the following perspectives.

First, WG affects an employee's self-efficacy and hope. Informal work-related social cues provided by WG represent individual opinions and others' perspectives on work-related information in the organization, serving as reference points and adjusting individuals' self-efficacy (23). Based on cognitive appraisal theory, when personal emotion is evoked by interpreting, appraising, or evaluating the relevant information, an individual will adopt a coping strategy to strengthen or erase the mental states of emotion (7, 14, 15). When gossipers interpreting social cues from idle talk, they engage in the social information process (13), leading them to make social comparisons between themselves and the gossip target (3) and gain or lose personal resources from the social information (24). For example, WPG enhances the reputation of the absent target by highlighting exemplary behaviors or praising persons in the organization. These positive social cues and information may evoke individual positive mental states of emotion (7, 15). To strengthen this positive mental state (14), employees who participate in WPG may regard gossip targets as role models for social learning and hope to mimic them to also reach positive achievements. Therefore, WPG enhances one's desires to improve capabilities and increases their motivation to attain desired outcomes (12). Although WPG participants consume their resources to interpret the social information cues, they also obtain more positive psychological resources of self-efficacy and hope. Thus, participating in WPG positively affects an individual's self-efficacy and hope of PsyCap to achieve positive outcomes.

Conversely, WNG refers to negative evaluation about the absent target's reputation. Kuo et al. (1) posited that work-related WNG can be a sign of an awareness that the WNG target does not reach the expectations of their work tasks assigned by an organization or supervisors. Thus, individuals engaging in such negative chatter imply that the gossip targets are inferior in their capabilities and behaviors when dealing with work-related duties. In other words, these negative evaluations imply that the gossip target needs to improve their capabilities to meet the task or challenge. Based on cognitive appraisal theory, these negative evaluations of a colleague may also evoke the gossiper's negative mental states when they interpreting, appraising, or evaluating the WNG information (7, 14, 15). Specifically, WNG implies that everyone is uncertain how his or her performance would be evaluated by others. Ashford (25) contended that individuals become more cautious and unconfident about how their behaviors or results will be evaluated by others under a high degree of uncertainty. Brady et al. (3) demonstrated that when there is an unknown range of performance evaluation, individuals adopt social comparisons to compare themselves with others when engaging in WNG. Therefore, due to the uncertainties of evaluation from others, a WNG gossiper is not sure if their capabilities are definitely better than the gossip target in the social comparison process. In other words, the WNG gossiper becomes anxious about his or her own capabilities with regard to others' perspectives. Moreover, WNG participants may have less confidence and experience more pressure to identify whether they could better complete the task assigned by their organization or supervisors due to the uncertainty of their performance when delivering others' negative evaluations; otherwise, they may fall victim to negative gossip (1). Therefore, WNG participants may experience more pressure at work and decreased self-efficacy and hope in work-related tasks due to fear of failure. Furthermore, research has demonstrated a strong negative direct effect of WNG on employees' self-efficacy and motivation for success (3, 26). Thus, WNG participants consume their resources to interpret WNG and also experience reduced self-efficacy and hope.

Second, WG may affect employee optimism and resilience. Studies have suggested that employees engage in WG to gain social resources (8). Gossip provides social support by establishing social bonds and trust relationships (22), which may influence individual optimism (27) and resilience (28). Thus, a social resource exchange may exist during WG. From the cognitive appraisal perspective (14), gossipers engaging in WPG may receive positive social information that yields more social support resources in the form of inspiration and energy to reach a positive mental state of emotion (29). Accordingly, individuals with more social support resources have more optimism towar life (30) and more resilience when facing difficulties (31). In contrast, WNG would evoke a individual negative mental state of emotion (15), Turner et al. (32) pointed out that participating in WNG may ruin relationships due to the negative information intended to depreciate others. Moreover, the gossiper may be regarded as a non-credible communicator if the information is rumored (33). Thus, when an individual with poor relationships is regarded as non-credible in the workplace, he or she may be ostracized by other colleagues and receive few social resources or less social support (34), which can ultimately diminish their optimism about work and reduce their resilience following a work setback.

Here we argue that WG may offer positive and negative social information cues for employees to adjust their attitudes and behaviors due to upward and downward comparisons, affecting individuals' self-efficacy and hope for positive outcomes. WG may also provide or block social resources that influence employees' optimism and resilience at work. Thus, this study proposes the following:

*Hypothesis 2a*: Participating in WPG is positively correlated with gossiper's PsyCap.
*Hypothesis 2b*: Participating in WNG is negatively correlated with gossiper's PsyCap.

### Relationship Between Psychological Capital and Mental Health

Positive psychological resources are the core construct of PsyCap (35), and individuals require these resources to maintain and develop their psychological well-being (36). Studies have identified that individuals with greater PsyCap experience less stress (37), anxiety (38), and depression (39). Moreover, researchers have determined that increases in individuals' PsyCap enhance the perception of well-being and reduce symptoms of mental illness (40). This study followed previous work to verify the relationship between PsyCap and MH and proposed the following:

*Hypothesis 3*: PsyCap is positively correlated with MH.

### Mediating Role of Psychological Capital

Furthermore, this study proposed that WG may affect MH through PsyCap. WG frequently occurs with informal social interactions when people can deliver or exchange social cues for informal social resources (8). Such exchanged informal social resources can result in personal resource spending on interpreting WG social cues (1), but they also confer the resource gains of social information and social support (8). Specifically, gossipers determine the reliability and value of WG from other employees' perspectives, which costs personal social and psychological resources and unscrambles the social cues from their own perspectives. When gossipers interpret WG from their unique perspective, they could acquire social and psychological resources that affect their PsyCap. However, participating in different types of WG would cause different results of the PsyCap dynamic.

WPG enhances gossipers' hope and self-efficacy by motivating them to reach similar achievements as those of the gossip target due to social comparison (3). Furthermore, WPG participants can gain a sense of trust and social support from others because of the positive information transmission (29), thereby enhancing individuals' optimism and resilience. Therefore, participating in WPG would have positive effects on gossiper's PsyCap. In contrast, WNG participation may reduce gossipers' self-efficacy and hope due to exposure of the negative social cues of fear of being negatively evaluated by others (25). In addition, when the negative evaluation of WNG is fake or exaggerated, the gossiper may affect the relationships among gossipers, gossipees, and gossip targets, which could also damage the gossiper's reputation or trustworthiness. Therefore, WNG gossipers may lose social resources and support from others after they speak ill of others, thereby affecting their optimism and resilience. Therefore, PsyCap is affected when individuals' self-efficacy, hope, optimism, and resilience change from participating in WG.

When employees' PsyCap is influenced by WG, it may continue to affect their MH. The conservation of resources theory suggested that individuals could acquire, maintain, and foster psychological resources from WG to prevent future resource depletion (36), implying that PsyCap is a key factor for individuals to maintain and protect their mental well-being from threat or loss (37), burnout (38), and illness (40). Here we further propose that when employee gaining PsyCap from engaging in WPG, there are indirect positive effects on gossipers' MH. In other words, PsyCap from WPG would serve as a positive influence on participants” MH, while participating in WNG would cause gossipers to lose PsyCap and indirectly and negatively affect gossipers' MH. Therefore, besides the direct effect of WG on PsyCap, we expect that WG may have indirect effects on participators' MH through PsyCap.

In summary, we predicted that PsyCap plays a mediator role in the relationship between WG and individuals' MH. Thus, this study proposed the following:

*Hypothesis 4a*: PsyCap mediates the positive relationship between WPG and MH.
*Hypothesis 4b*: PsyCap mediates the negative relationship between WNG and MH.

### Moderating Effect of Developmental Job Experience

As opposed to organizations' and managers' attempts to minimize the influence of WG on employee (41), this study seeks to use the function of work itself to help employees cope with WG. DJE is a process of self-development in which employees learn, growth, and improve their capabilities through effectively accomplishing their role task or achieving their goals (9). We choose three suitable dimensions identified by McCauley et al. (9) for staff members (rather than top-level leadership) to measure DJE. The first is unfamiliar responsibilities that require an employee to take on different or new roles and tasks and are often assigned when the organization implements a job transition. The second is high-level responsibility for assigned tasks that are highly visible to management and substantially affect high-level stakeholders or the organization. The third is working across boundaries that an employee with little formal authority may be asked to coordinate with other internal peers, departments, and supervisors or individuals external to the organization. Previous studies have reported that DJE has significant effects on individuals' information processing (42). Therefore, we expect that the interaction between WG and DJE would have a moderating effect on PsyCap and MH.

WG is a type of informal information resource with uncertain reliability and validity (1, 8) that might interact with DJE. For instance, employees with relatively high boundaries of DJE have a superior background in interacting with others and processing ambiguous information (42) and are better able to judge WG reliability and accuracy. Furthermore, an employee with a higher unfamiliar responsibilities of DJE has higher comprehensive abilities, interpersonal capabilities, and adaptability (10), which could help that individual to view gossip from a systematic perspective (43). Moreover, employees with significant high-level responsibility of DJE exercises would accomplish their role tasks with this in full consideration. As a result, they would view WG more critically and identify underlying causes and consequences (44) rather than accepting it at face value.

When employees with high DJE participate in positive gossip, they critically think about the reasons for the gossip targets' positive outcomes (42) and learn from their successes. Therefore, they would obtain more self-efficacy, hope, and motivation from WPG if they have high DJE. Such employees thus interpret WPG as a valuable learning resource that provides psychological support to help them acquire higher achievement. However, employees with lower DJE obtain fewer resources to learn from WPG. That is because that lower DJE employees take WPG at face value, they lack sufficient experience to excavate the deeper meaning in WPG about one's achievement. Similarly, when employees with high DJE engage in WNG, they critically think about the underlying causes (42, 43) of mistakes and how to correctly view and treat negative results realized by the gossip target. WNG thus provides more optimism and resilience for those with high DJE, helping them learn from and avoid the negative gossip targets' failures. In other words, employees with high DJE interpret WNG as a warning to avoid making the same mistakes, or they seek resources to overcome the negative results. In contrast, employees with lower DJE lack sensitivity to these negative results as a sign to consider making the same mistake and thus receive few resources for facing the same problems. Moreover, employees with lower DJE may even spend resources to erase the fear of being next negative gossip target if they do not achieve the expected task results. Thus, the following hypothesis is proposed:

*Hypothesis 5a*: DJE moderates the relationship between WPG and PsyCap such that the effect of WPG on PsyCap is stronger with higher DJE.
*Hypothesis 5b*: DJE moderates the relationship between WNG and PsyCap such that the effect of WNG on PsyCap is stronger with lower DJE.

Based on the discussion of the relationships among WG, PsyCap, MH, and DJE, the present study predicted that the moderating effect of DJE has far reaching effects on employees' MH through PsyCap, which means that DJE moderates the indirect effect of WG on MH through PsyCap. Thus, this research further proposes a moderated mediation model as follows:

*Hypothesis 6a*: DJE moderates the relationship between WPG and MH through PsyCap such that the effect of WPG on MH is stronger with higher DJE.
*Hypothesis 6b*: DJE moderates the relationship between WNG and MH through PsyCap such that the effect of WNG on MH is stronger with lower DJE.

## Methods

### Participants and Procedures

Participants were full-time employees working in the same company in the tourism industry in Taiwan. Data were collected with anonymous paper questionnaires with supervisor cooperation at a travel agency in Taiwan. A collection approach of three waves over 3 months was adopted to minimize the single time-point method bias (45). Each participant was assigned a random identity code for their questionnaires. Instructions and return envelopes were also provided to participants. Furthermore, researchers provided an NT$150 gift voucher to participants who completed all items in the three waves of data collection to increase their motivation. At Time 1, researchers distributed 300 copies of Wave 1 questionnaires and received 262 responses. One month later, researchers sent out Wave 2 questionnaires to participants who had answered questionnaires in Wave 1 and collected 239 responses. A month later, researchers distributed the final wave of questionnaires to participants who had completed Waves 1 and 2. This study collected 222 responses (74.00% response rate). The sample demographics were as follows: 167 women (75.20%) and 55 men (24.80%), mean age of 36.82 (*SD* = 7.54), the majority held a bachelor's degree (83.80%), the average employee average tenure was 10.24 years (*SD* = 7.17), 137 were married (61.70%), and 146 participants were staff (65.80%).

### Measures

#### WG (Wave 1)

WG was measured in Wave 1 using a 12-item questionnaire on work-related gossip, with six items for WPG and six for WNG developed by Kuo et al. (1). A sample item for WPG is “Have you recently gossiped about a colleague's excellent job performance?” A sample for WNG is “Have you recently gossiped about a colleague's carelessness and poor work engagement?” All items in this research were scored using six Likert-type response options ranging from 1 (*strongly disagree*) to 6 (*strongly agree*).

#### DJE (Wave 1)

This study assessed DJE with the 20-item Job Challenge Profile (46) in Wave 1. A sample item is “This job asks you to manage something with which you are unfamiliar.”

#### PsyCap (Wave 2)

This study measured PsyCap using the 24-item Psychological Capital Questionnaire (5) in Wave 2. A sample item is “If I find myself in a jam at work, I could think of many ways to get out of it.”

#### MH (Wave 3)

The 12-item version of the General Health Questionnaire Scale (47) was used to assess MH in Wave 3. A sample item is “Have you been able to face your problems?” Furthermore, this study used a scoring system wherein a higher score implying better MH.

#### Control Variables (Wave 1)

The study controlled for several factors to minimize the effect of demographic variables (gender, age, educational level, job tenure, marital status, and position). We also controlled for job stress, with a single item from the study by Elo et al. (48), as it may be related to PsyCap (49) and MH (50).

## Results

Table 1 presents the means, standard deviations, bivariate correlations, and Cronbach's alpha values. The results indicates that WPG was positively correlated with MH (*r* = 0.15, *p* < 0.05) and PsyCap (*r* = 0.24, *p* < 0.001). PsyCap was significantly positively correlated with MH (*r* = 0.54, *p* < 0.001). WNG negatively correlated with MH (*r* = −0.24, *p* < 0.001) and PsyCap (*r* = −0.18, *p* < 0.01). The correlation coefficient results initially support H1, H2, and H3.

**Table 1:** Variables means, standard deviations, reliabilities, and correlations (*N* = 222).

**Variables**	***M***	***SD***	**1**	**2**	**3**	**4**	**5**	**6**	**7**	**8**	**9**	**10**	**11**	**12**
1. Gender^†^	0.25	0.43												
2. Age	36.82	7.54	−0.01											
3. Education^†^	2.91	0.39	0.11	−0.17^**^										
4. Tenure (years)	10.24	7.17	0.02	0.81^***^	−0.14^*^									
5. Marital status^†^	0.38	0.49	0.15^*^	0.32^***^	−0.01	0.22^**^								
6. Position^†^	1.44	0.69	0.21^**^	0.48^***^	0.03	0.54^***^	0.24^***^							
7. Job stress	4.23	1.15	0.04	0.05	−0.09	0.06	−0.04	0.03						
8. WPG	4.52	0.68	0.12	0.00	0.08	0.04	−0.02	0.16^*^	−0.05	(0.70)				
9. WNG	3.28	1.01	−0.09	0.09	−0.09	0.13	−0.01	0.10	−0.02	0.41^***^	(0.83)			
10. PsyCap	4.45	0.46	0.18^**^	0.12	−0.03	0.16^*^	0.19^**^	0.18^**^	0.05	0.24^***^	−0.18^**^	(0.90)		
11. MH	4.28	0.58	0.12	0.06	0.09	0.04	0.14^*^	0.16^**^	−0.29^***^	0.15^*^	−0.23^***^	0.54^***^	(0.83)	
12. DJE	3.88	0.55	0.17^*^	0.05	−0.01	0.11	0.02	0.18^**^	0.14^*^	0.35^***^	0.16^**^	0.24^***^	−0.09	(0.78)

*Cronbach's alpha, α is presented in the diagonal*.

*Gender (0, female; 1, male); Education [1, junior high school or below; 2, (vocational) senior high school; 3, bachelor's degree; and 4, master or above]; Marital status (0, single; 1, married); Position (1, staff; 2, junior manager; 3, mid-level manager; and 4, senior manager)*.

* *p < 0.05*,

** *p < 0.01*,

*and*

*** *p < 0.001*.

### Model Analyses

The study used confirmatory factor analysis (CFA) in AMOS 21 software with parceling rules (51) to test the fit of the hypothesized model. The overall CFA results indicated that the hypothetical five-factor model demonstrated a good fit with the data [χ^2^ = 274.43, comparative fit index (CFI) = 0.90, incremental fit index (IFI) = 0.91 root mean square error of approximation (RMSEA) = 0.08]. This study also tested four other alternative-factor models. Other models' goodness-of-fit statistical results indicated that the hypothetical five-factor model had a better fit for data (Table 2). In sum, model comparison results suggested that the hypothetical constructs were a good fit for statistical significance.

**Table 2:** Results of confirmatory factor analyses of the measures (*N* = 222).

**Model**	**Factors**	**χ^2^**	**df**	**Δχ^2^/df**	**CFI**	**IFI**	**RMSEA**
Hypothetical model	5 factors	274.43	109		0.90	0.91	0.08
Model 1	4 factors	323.16	113	12.18	0.88	0.88	0.09
Model 2	3 factors	567.24	116	41.83	0.73	0.74	0.13
Model 3	2 factors	692.25	118	46.42	0.66	0.67	0.15
Model 4	1 factor	1,216.07	119	121.61	0.35	0.37	0.20

*Hypothetical model (5 factors: workplace positive gossip; workplace negative gossip; psychological capital; mental health; developmental job experience)*.

*Model 1 (4 factors: workplace positive gossip and workplace negative gossip merged; psychological capital; mental health; developmental job experience)*.

*Model 2 (3 factors: workplace positive gossip, workplace negative gossip and developmental job experience merged; psychological capital; mental health)*.

*Model 3 (2 factors: workplace positive gossip, workplace negative gossip and developmental job experience merged; psychological capital and mental health merged)*.

*Model 4 (1 factor: all variables are merged into one factor)*.

### Hypothesis Testing

PROCESS macro software (52) is used to analyze complicated research models. In this study, we used three existing model syntaxes of constructions (mediation, moderation, and moderated) in the PROCESS macros to perform hypothesis testing.

Hypotheses 1a and 1b stated that WG would correlate with MH. The results of simple linear regression test in PROCESS macro software are presented in Table 3. The study controlled for demographic variables and job stress. WPG positively correlated with MH (β = 0.21, *p* < 0.001), and WNG negatively correlated with MH (β = −0.20, *p* < 0.001), which supported Hypotheses 1a and 1b. Similarly, Hypotheses 2a and 2b were also supported by positing that the relationship between WPG and PsyCap was positively correlated (β = 0.25, *p* < 0.001) and WNG and PsyCap were negatively correlated (β = −0.16, *p* < 0.001) As shown in Table 3, the findings also supported Hypothesis 3, which proposed that PsyCap has a positive relationship with MH. When this study regressed PsyCap on MH, we observed a positive relationship between PsyCap and MH (β = 0.69, *p* < 0.001).

**Table 3:** Regression results for simple mediation (*N* = 222).

**Variables**			***B***	***SE***	***t***	***p***
**Direct and total effects**						
**WPG** ** → MH**			0.21	0.06	3.68	0.000
**WNG** ** → MH**			−0.20	0.04	−5.09	0.000
**WPG** ** → PsyCap**			0.25	0.05	5.33	0.000
**WNG** ** → PsyCap**			−0.16	0.03	−5.08	0.000
**PsyCap** ** → MH**			0.69	0.07	9.98	0.000
**PsyCap** ** → MH** (controlling for WPG and WNG)			0.62	0.07	8.46	0.000
**WPG** ** → MH** (controlling for PsyCap)			0.06	0.05	1.09	0.28
**WNG** ** → MH** (controlling for PsyCap)			−0.10	0.04	−2.76	0.006
	**Value**	***SE***	**LL 95% CI**		**UL 95% CI**	
**Indirect effect and significance of WPG on MH**						
Bootstrap	0.15	0.04	0.09		0.24	
**Indirect effect and significance of WNG on MH**						
Bootstrap	−0.10	0.02	−0.15		−0.06	

*Standardized regression coefficients are reported. Bootstrap sample size = 50,000*.

*LL, lower limit; CI, confidence interval; UL, upper limit*.

This study further used mediation testing in PROCESS macro software to test the mediating effect of Hypotheses 4a and 4b, and the results illustrated that PsyCap mediates the relationship between WG and MH. As mentioned previously, WG, PsyCap, and MH were positively (WPG) and negatively (WNG) correlated. Therefore, we regressed WPG on MH while controlling for PsyCap. The results revealed that the standardized coefficient was significantly lower (β = 0.06, *p* > 0.05). Moreover, the standardized coefficient significantly decreased for WNG (β = −0.10, *p* < 0.01). The aforementioned evidence initially supported that PsyCap had a mediating effect on the relationship between WG and MH. We also estimated indirect effects using a bootstrap approach with 95% confidence intervals (CIs). The results in Table 3 indicate that PsyCap mediated the relationship between WG and MH (β = 0.15 for WPG, β = −0.10 for WNG). Ultimately, the results of bootstrap testing revealed that the 95% CIs did not contain zero for WPG (0.09, 0.24) or WNG (−0.15, −0.06) analyses. Overall, Hypotheses 4a and 4b were supported.

**Figure 1:**
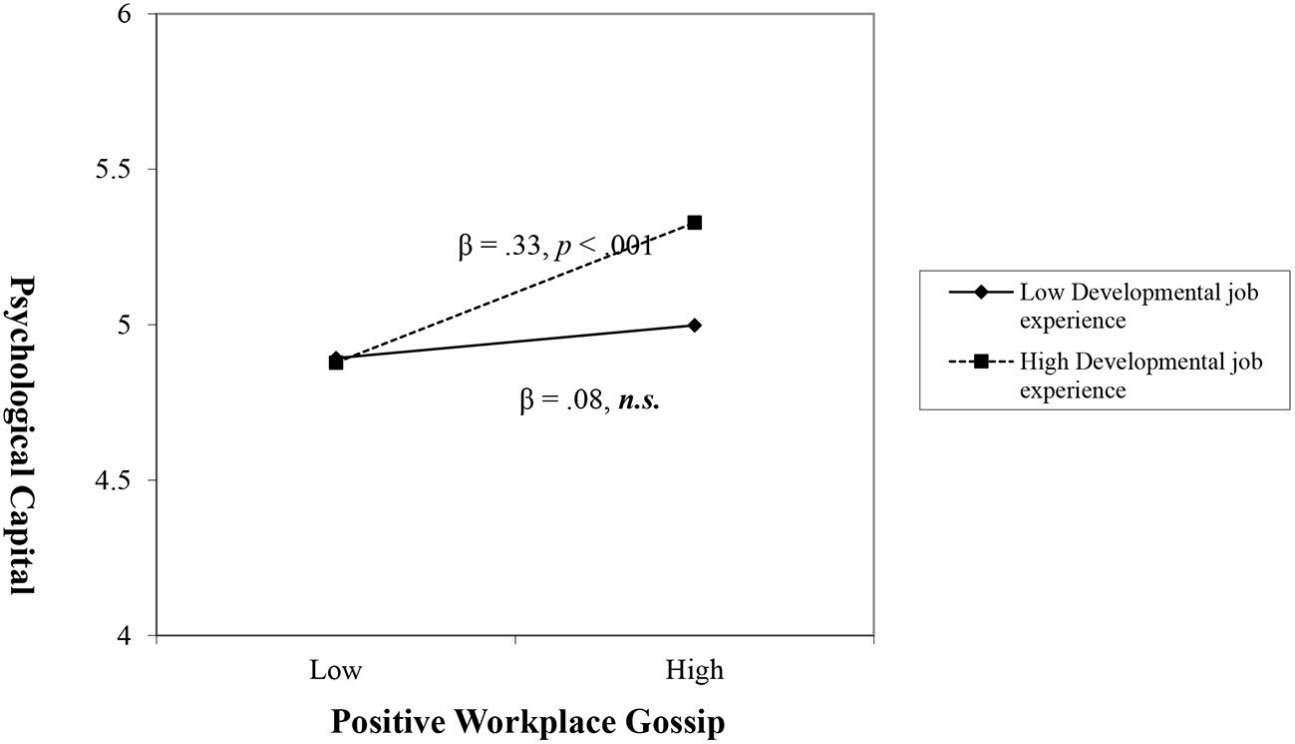
Moderating effect of developmental job experience on the relationship between workplace positive gossip and psychological capital.

**Figure 2:**
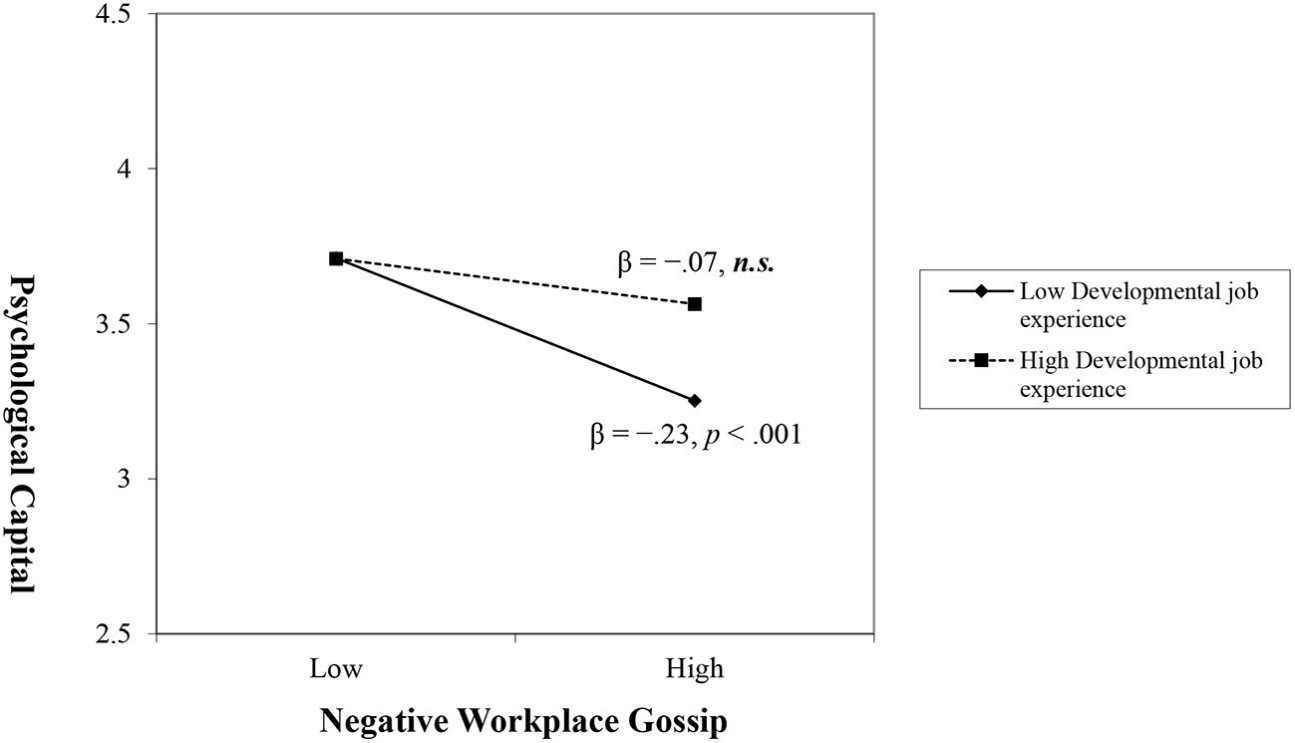
Moderating effect of developmental job experience on the relationship between workplace negative gossip and psychological capital.

Hypotheses 5a and 5b proposed that DJE moderates the relationship between WG and PsyCap. Table 4 presents the results of a simple moderating effect using the moderation test in the PROCESS macro software. The interaction of DJE and WG was statistically significant for both WPG (β = 0.23, *p* < 0.001) and WNG (β = 0.14, *p* < 0.01). The 95% CIs showed that neither *WPG* × *DJE* (0.12, 0.36) nor *WNG* × *DJE* (0.05, 0.24) contained zero. Furthermore, this study examined conditional effects by dividing DJE into three groups at the mean and one standard deviation above and below the mean to represent low-, medium-, and high-level moderators. The results of the conditional effects of each group on DJE (Table 4) show that the effect of WPG on PsyCap was positively statistically significant at the high level (β = 0.33, *p* < 0.001) but not at the low level (β = 0.08, *p* > 0.05). The moderated effect of WNG on PsyCap was significant at the low level (β = −0.23, *p* < 0.001) but not at the high level (β = −0.07, *p* > 0.05). Figures 1, 2 present the moderating effects of DJE on the relationship between WG and PsyCap. Hypotheses 5a and 5b were supported by our findings.

**Table 4:** Regression results for moderation (*N* = 222).

***Simple moderation results for PsyCap***					
**Values of moderators in** ***Simple moderated effect***	**Conditional effect**	***SE***	**Boot LL 95% CI**	**Boot UL 95% CI**	***p***
DJE × WPG	0.23	0.06	0.12	0.36	0.000
−1 *SD* (3.33)	0.08	0.06	−0.04	0.20	0.19
*M* (3.88)	0.20	0.05	0.12	0.30	0.000
+1 *SD* (4.42)	0.33	0.06	0.23	0.45	0.000
DJE × WNG	0.14	0.05	0.05	0.24	0.003
−1 SD (3.33)	−0.23	0.04	−0.32	−0.16	0.000
M (3.88)	−0.15	0.03	−0.22	−0.09	0.000
+1 SD (4.42)	−0.07	0.04	−0.16	0.02	0.088
***Moderated mediation results for MH (WPG)***					
**Values of moderators in** ***Moderated mediation effect***	**Conditional indirect effect**	***SE***	**Boot LL 95% CI**	**Boot UL 95% CI**	
−1 SD (3.33)	0.06	0.48	−0.04	0.15	
M (3.88)	0.14	0.04	0.07	0.22	
+1 SD (4.42)	0.22	0.05	0.14	0.33	
**Index of moderated mediation**	**Index**	***SE***	**Boot LL 95% CI**	**Boot UL 95% CI**	
DJE (WPG)	0.15	0.05	0.06	0.27	
***Moderated mediation results for MH (WNG)***					
−1 SD (3.33)	−0.16	0.03	−0.23	−0.11	
M (3.88)	−0.10	0.02	−0.15	−0.07	
+1 SD (4.42)	−0.05	0.03	−0.11	0.01	
**Index of moderated mediation**	**Index**	***SE***	**Boot LL 95% CI**	**Boot UL 95% CI**	
DJE (WNG)	0.10	0.04	0.03	0.18	

*Standardized regression coefficients are reported. Bootstrap sample size = 50,000*.

*LL, lower limit; CI, confidence interval; UL, upper limit*.

Hypotheses 6a and 6b stated that DJE moderates the mediated relationship between WG and MH through PsyCap. Hypotheses 6a and 6b were examined using the moderated mediation model test in the PROCESS software, which assessed the conditional indirect effect of DJE at different levels. Table 4 presents the moderated mediation results for MH. The results indicated that the index of conditional indirect effects of moderated mediation (β = 0.15 for WPG, β = 0.10 for WNG) were significant in WPG (0.06, 0.27) and WNG (0.03, 0.18), as they did not contain zero at the 95% CI level. We also tested the moderated mediation effect at all levels of means in two types of WG (Table 4). As for WPG, the moderated mediation effect was significant at a high level (β = 0.22) for the 95% CIs (0.14, 0.33); WNG was significant at a low level (β = −0.16), with 95% CIs not containing zero (−0.23, −0.11). Hence, Hypothesis 6a and 6b were supported.

## Discussion

This study was designed to explore the effect of WG on participators' MH by assessing PsyCap as the underlying mediated mechanism and investigated the moderating effect of DJE. This study conducted three waves of data collection with 222 full-time employees from a Taiwanese tourism company who were invited to participate. The results revealed that WPG and WNG positively and negatively affected participators' MH, respectively. The two relationships were differentially mediated by PsyCap. Furthermore, employees with higher DJE exhibited greater capacity to positively interpret WPG and cope with WNG, which ultimately affected individual's PsyCap and had indirect effects on individual's MH. Therefore, DJE played a moderating role among WG, PsyCap, and MH.

### Theoretical Implications

The study makes four contributions to the literature on WG. First, this study determined that WG would affect individuals' psychological health, which have rarely been investigated. Moreover, we observed that WPG enhances MH, whereas WNG has a negative impact. The results also showed that both types of WG should be assessed (2, 3), and although WPG is a deviant behavior, it has potential for serving a social function at work.

Second, the present study proposed an alternative psychological mechanism of PsyCap to explain the underlying effects of WG on participators' MH. Although previous studies have described possible mechanisms from cognitive, affective, emotional, or psychological perspectives (4), few studies have conceived of WG as a personal resource that may affect individual MH from a psychological resources perspective. The findings suggested that individuals engaging in different types of WG may cause different dynamic changes in PsyCap based on their interpretation of WG social cues (1), which might differentially affect gossipers' MH. Furthermore, our results suggest that engaging in WG can be a channel for individuals to exchange personal resources, and further explained how and why WG can be interpreted as a psychological resource to affect gossiper's MH through PsyCap.

Third, this study identified the boundary conditions for the effect of WG on participators' MH. The results highlight an important moderator of both WPG and WNG. Our findings of moderated effects reaffirm prior studies demonstrating that employees with high DJE tend to have a more sensitive and systematic approach to social cue interpretation (9, 10). Specifically, our results showed that the direct effects of WPG on PsyCap and indirect effects of WPG or MH through PsyCap would be strengthened for employees with high DJE. Moreover, an employee with significant DJE would attenuate the negative effects of WNG on PsyCap or MH through PsyCap, which means that high DJE would provide a coping mechanism for them to mitigate the negative effects of WNG engagement. Thus, this study identified that DJE could interact with individuals' social information processing (13) and demonstrated the individual differences of DJE during social cue interpretation (42). In summary, this study identified a valuable boundary condition in the relationships among WG, PsyCap, and MH.

### Practical Implications

In practical terms, WG frequently occurs in daily conversations (19), so it is important for employees, managers, and organizations to realize its meaning in organizational settings (1). The findings of the present study suggest the following implications.

Firstly, as indicated in previous studies, WG cannot be viewed only as deviant behavior but as one that offers the positive functions of enhancing information exchange in organizations, facilitating friendships, or providing entertainment (2, 11). Specifically, WPG participation positively correlates to participators' PsyCap and MH (through PsyCap), whereas engaging in WNG is negatively related to gossipers ‘PsyCap and MH (through PsyCap). Thus, the first intervention for managers is to guide employees to talk about more positive gossip when they engage in idle talk behaviors. For example, managers can encourage employees to discuss others' positive evaluations during break times or post bulletin board notices in the break room. Moreover, managers could also offer public praise for outstanding employee performance (53), which could provide a topic for informal chats. However, our results do not indicate that managers should intentionally create opportunities for positive gossip to improve employees' MH. While managers cannot completely prevent WNG (21, 26, 29), they can minimize its occurrence (41). Liu et al. (21) suggested that organizations can establish a zero-tolerance organizational culture or issue rules and norms for WNG. Moreover, managers can educate employees (19) about why and how negative gossip can diminish PsyCap and exacerbate MH issues.

Secondly, this research revealed the moderating effect of DJE, which can help individuals intensify the positive effects of WPG and cope with the negative effects of WNG. As for interventions, managers can assign extra challenge tasks or implement job transitions that help employees develop new skills (46). Managers could also assign employees a highly interactive recurrent task in cooperation with peers, supervisors, and colleagues in other departments or provide task opportunities to deal with government officials or suppliers. By meeting these challenges, employees could enhance their DJE. Organizations could also communicate the meaning of DJE by encouraging employees to voluntarily accept challenging tasks (10).

### Limitations and Future Directions

This study also has limitations. First, due to convenience sampling, this study only tested one specific industry. Hence, this study did not test the effects of industry differences on hypotheses. The service industry, like the tourism industry, provides more opportunities for employees to interact at work or interact with suppliers, customers, or other companies outside the organization than the manufacturing industry (54). Therefore, the tourism industry may have a higher frequency of WG and higher perception and sense of DJE than manufacturing. In the future, researchers could investigate industry differences in WG and DJE to provide holistic and robust perspectives.

Second, we considered gossiper's PsyCap but neglected to control for other relevant contextual variables such as other job resources and job demands (8, 21). Furthermore, we may have overlooked the effects of some personality dispositions (1), a consideration that may have yielded stronger evidence for the predictive power of gossip. Future work should consider controlling for these variables to yield more robust results regarding the effects of WG on employees.

Third, this study only stressed the importance of the moderated effect of DJE, indicating its different functions when coping with the two types of WG. Future investigations can examine more boundary conditions or other multilevel perspectives to clarify other moderating effects of WG on employee attitudes and behaviors.

Forth, this research model only tested the individual-level model. Others have indicated that cross- or multilevel approaches of WG are alternative perspectives for investigating organizational phenomena (1) and suggested that WG can create a social context for employees. Thus, future study can collect and analyze WG from a multilevel perspective to explore the organizational effect on employees' outcomes.

## Conclusion

This study indicated the effects two types of WG on participators' MH and highlighted the mechanism of the psychological process. WPG (WNG) can be a positive (negative) trigger to enhance (reduce) employee psychological resources. Furthermore, these results revealed a moderating effect on employees with high DJE who have better capabilities to absorb the positive energy of WPG and cope with WNG.

## Data Availability Statement

The raw data supporting the conclusions of this article will be made available by the authors, without undue reservation.

## Author Contributions

SC, C-CK, and VK contributed to conception and design of the study. H-CC organized the database. SC and M-CL performed the statistical analysis. SC wrote the first draft of the manuscript. All authors revamped sections of the manuscript and contributed to manuscript revision, read, and approved the submitted version.

## Funding

The work was funded by Ministry of Science and Technology, Taiwan 108-2410-H-004-090.

## Conflict of Interest

The authors declare that the research was conducted in the absence of any commercial or financial relationships that could be construed as a potential conflict of interest.

## Publisher's Note

All claims expressed in this article are solely those of the authors and do not necessarily represent those of their affiliated organizations, or those of the publisher, the editors and the reviewers. Any product that may be evaluated in this article, or claim that may be made by its manufacturer, is not guaranteed or endorsed by the publisher.
